# Region-based analysis in genome-wide association study of Framingham Heart Study blood lipid phenotypes

**DOI:** 10.1186/1753-6561-3-s7-s127

**Published:** 2009-12-15

**Authors:** Jennifer L Asimit, Yun Joo Yoo, Daryl Waggott, Lei Sun, Shelley B Bull

**Affiliations:** 1 Samuel Lunenfeld Research Institute of Mount Sinai Hospital, 60 Murray Street, Box 18, Toronto, Ontario M5T 3L9, Canada; 2 Dalla Lana School of Public Health, University of Toronto, 155 College Street, Toronto M5T 3M7, Canada; 3 Department of Statistics, University of Toronto, 100 St. George Street, Toronto M5S 3G3, Canada

## Abstract

Due to the high-dimensionality of single-nucleotide polymorphism (SNP) data, region-based methods are an attractive approach to the identification of genetic variation associated with a certain phenotype. A common approach to defining regions is to identify the most significant SNPs from a single-SNP association analysis, and then use a gene database to obtain a list of genes proximal to the identified SNPs. Alternatively, regions may be defined statistically, via a scan statistic. After categorizing SNPs as significant or not (based on the single-SNP association *p*-values), a scan statistic is useful to identify regions that contain more significant SNPs than expected by chance. Important features of this method are that regions are defined statistically, so that there is no dependence on a gene database, and both gene and inter-gene regions can be detected. In the analysis of blood-lipid phenotypes from the Framingham Heart Study (FHS), we compared statistically defined regions with those formed from the top single SNP tests. Although we missed a number of single SNPs, we also identified many additional regions not found as SNP-database regions and avoided issues related to region definition. In addition, analyses of candidate genes for high-density lipoprotein, low-density lipoprotein, and triglyceride levels suggested that associations detected with region-based statistics are also found using the scan statistic approach.

## Introduction

Definition of an appropriate unit of gene function has been identified as a fundamental issue in genetic association analysis using high-dimensional single-nucleotide polymorphism (SNP) data [1]. On one hand, the use of SNPs selected to capture variation across the whole genome may lend itself to treating a single SNP as the unit of analysis for false-positive error control. On the other hand, allocating SNPs into regions and treating the region as the unit of analysis can substantially reduce the dimensionality problem at the genome level, and is natural when the region corresponds to a candidate gene. Neale and Sham put forth an eloquent argument for such a gene-based approach [2]. Given that a set of SNPs deemed to be relevant to a particular candidate region can be identified, the issue of how to evaluate genetic association for the candidate gene/region remains. Application of test statistics for multiple SNP markers within a chromosomal region may help address the problem of multiple testing by increasing the power to detect associations and/or reducing the number of tests conducted.

Scan statistics based on single-SNP tests have been proposed to identify genomic regions associated with disease [3,4], whereas others consider a class of test statistics with small degrees of freedom (***df***) that combine information across a set of SNP markers within an identified region [5]. A multi-locus regression-based test statistic that simultaneously tests for main effects of all the SNP loci within a region, ignoring haplotype phase, can be more powerful than haplotype analysis [6] because it allows for association across multiple markers but does not "spend" ***df ***on rare haplotypes. At the other extreme, the results of multiple single ***df ***tests of SNPs within a candidate region require adjustment for multiple testing. A number of authors compared various test statistics, mainly in the case-control setting, finding that relative performance depends on the density and the correlation structure of the SNPs within a region, the selection criteria and the number of SNP markers, the placement and the number of liability/causal SNPs within a region, as well as on allele frequencies and the presence of allelic heterogeneity.

In this contribution, we apply two region-based approaches to a genome-wide association study (GWAS) analysis of blood lipid measures taken in members of Offspring Cohort and Generation 3 Cohort of the Framingham Heart Study (FHS). Initially, we tested each of the 550 k SNPs from the Affymetrix array datasets, one at a time. In an alternate approach, we applied scan statistics based on the single-SNP *p*-values to identify and test genomic regions simultaneously. Taking a more conventional approach, we also used external information from the UCSC gene database [7] to define gene and inter-gene regions corresponding to single SNPs with small *p*-values. Within the defined genomic regions, we then applied region-based test statistics using multiple linear regressions of sets of SNPs. We compare the two analytic strategies in GWAS with respect to the SNPs and the regions detected, and also compare the association test results in a set of regions defined by candidate lipid genes.

## Methods

### FHS data

We analyzed the Genetic Analysis Workshop 16 FHS Offspring Cohort (*n *= 2584) and Generation 3 Cohort (*n *= 3811) using the SNP genotypes from GeneChip Human Mapping 500 k Array and 50 k Human Gene Focused Panel and the blood lipid phenotypes. All family members within these cohorts who had been genotyped and phenotyped were included in the analysis.

### Definition of phenotypes

Fasting total cholesterol, high-density lipoprotein (HDL) cholesterol and triglycerides (TG) were measured at up to four exams for the Offspring Cohort and at one exam for the Generation 3 Cohort. Low-density lipoprotein (LDL) cholesterol was calculated using the Friedewald formula (Total = HDL + LDL + TG/5) for each measurement. For the patients on lipid lowering medication, the actual total cholesterol and TG values were imputed following the method of Kathiresan et al. [8]. Imputation models were obtained separately by sex, and the sequential imputation process was performed separately within age-sex subgroups (10-year groups). TG values were log-transformed. The phenotype values were averaged over the multiple exams, as were the corresponding covariate values. We adjusted the mean HDL, mean LDL, and mean TG values for the averaged covariates using linear regression and treated the residuals as the phenotype values for the genotype-phenotype analysis. Two covariate models were used for the adjustment of phenotypes, separately by sex: Model 1: age and age^2^, and Model 2: age, age^2^, body mass index, alcohol intake, and cigarette smoking.

### Quality control of SNP genotype data

Quality control was completed using the computer programs PLINK [9] and Eigenstrat [10]. SNPs were filtered at a minor allele frequency <1%, Hardy-Weinberg equilibrium <10^-10 ^and call rate <90%. Samples were filtered at a call rate <90%. There were no outliers for exclusion, as determined using Eigenstrat.

### Individual level single-SNP association analysis

Linear regression of each of the residual phenotypes (Mean-HDL, Mean-LDL, Mean-TG) was performed using PLINK for each of the 550 k SNPs that passed filtering, based on a simple regression of additive SNP coding, including all individuals and ignoring familial correlation. Departures from the expected asymptotic distributions were assessed via quantile-quantile (Q-Q) plots for each of the phenotypes.

### Region identification and testing via scan statistics

The scan statistic approach identifies regions of significant SNPs and tests for regional significance [3]. It requires the SNP position and the *p*-value for association at that position. A group of SNPs tends to be identified as a region if there is statistical evidence of clustering of positions and of small *p*-values. The locations of SNPs along a chromosome are assumed to follow a Poisson process. To detect regions of association, the original Poisson process is partitioned into two independent Poisson processes, according to a chosen *p*-value threshold level. The resulting sets of SNP locations are both Poisson processes, with rates proportional to the original process. When the assumption of independent processes is violated, some regions may be detected solely because of their marker correlation structure, so to reduce the correlation among SNPs, we pruned the data by choosing tagSNPs with a pair-wise linkage disequilibrium (LD) *R*^2 ^threshold less than 0.5 [4].

Using the statistical package R, we identified regions of association by evaluating windows along the chromosome including varying numbers of SNPs, and tested for region-level significance. The regional *p*-value is the probability of observing the same number of significant markers over a distance as short as or shorter than observed. The scan statistic is simply the distance spanned by the group of markers of interest, i.e., the sum of inter-marker distances. Under Poisson process assumptions of independently identically distributed exponential inter-SNP distances, the scan statistic follows a gamma distribution, so that the probability of a high association cluster is a gamma cumulative distribution function. If this observed regional probability is smaller than a pre-specified significance criterion, then the group of markers is identified as a cluster of significant associations not likely to occur simply by chance. Genome-wide regional *p*-values were calculated empirically, using 10,000 permutations of the tag-SNP *p*-values across positions. In each permutation we kept the top *n *regions, where *n *is the number of identified regions in the original analysis [4].

### Region identification and testing via database-defined regions

Using the UCSC database, a list of regions meeting genome-wide criteria for significance (*p *< 10^-4^) was formed from the single-SNP tests. If a SNP was within ± 5 kb of a gene, then the assigned gene region was the gene endpoints ± 5 kb. Otherwise, the SNP position ± 5 kb was classified as an inter-gene region. In each of the gene and inter-gene regions thus defined, we performed region-based analyses using multi-variable regression of *k *SNPs within the defined region using the generalized estimating equations (GEE) robust variance to account for familial correlation, and the linear regression model: E(residual lipid phenotype) = *α *+ *β*_1 _*x*_*G*1 _+ *β*_2 _*x*_*G*2 _+ ... + *β*_*k*_*χ*_*Gk*_. For test statistics, we calculated the global *k ****df ***test (Hotelling's test), the Schaid test (1 ***df ***linear combination of SNP-specific test statistics; [5]), and the James min *P *test (correlation adjusted minimum *p*-value; [11]). To address SNP collinearity and reduce dimensionality, we repeated these analyses using principal components constructed from within-region SNPs [12].

## Results and discussion

Markers from the 500 k chip, pruned for LD (*R*^2 ^< 0.5), were used as input to the scan statistic analysis. The proportion of markers retained per chromosome ranged from 36 to 52%, with a mean of 40%. We specified a SNP *p*-value threshold of 0.01 and a regional threshold of 0.001. We categorized a scan statistic region as a gene region if it overlapped with a defined gene region (± 5 kb), and called the remaining regions non-gene regions. For HDL, 135 gene and 105 non-gene regions were detected genome-wide, with similar proportions for LDL and TG (133/110 and 100/104 for gene/non-gene, respectively).

By design, the scan statistic can detect regions with multiple SNP associations or regions with LD, and is expected to fail to detect isolated SNPs. In order to determine how many single-SNP associations we may have missed, we compared the scan statistic regions with a list of single SNPs with *p*-values < 10^-4^. With this threshold, there were 344 to 400 SNPs for each of the three phenotypes, of which 75 to 80% were not included within the scan statistic regions, and conversely 60 to 66% of the regions did not contain any of these SNPs. Detailed results for HDL are provided in Table 1.

**Table 1:** Comparison of scan statistic regions with single-SNP tests for HDL having *p*-values < 10^-4^

	Scan statistic regions			
			
Single-SNP	Non-gene	Gene	SNPs missed by scan statistic regions	SNP totals
Inter-gene SNP	29	18	172	219
Within-gene SNP	0	35	146	181
Total no. SNPs	29	53	318	400

In a comparison of the scan statistic regions and the SNP-database regions for each of the phenotypes, approximately half of the genome-wide significant scan statistic regions do not overlap with the SNP-database regions, and are novel (Table 2). Defining the regions statistically avoids the problem of *ad hoc *region definitions. On the other hand, gene-based regions reflect prior knowledge and biological structure.

**Table 2:** Comparison of scan statistic regions with SNP-database regions defined from single-SNP tests for HDL having *p*-values < 10^-4^

	SNP-database region			
			
Scan-statistic region	Inter-gene	Within-gene	Regions detected only by scan statistic	Total no. regions
Non-gene scan statistic	33 (8)^a^	0	72 (12)	105 (20)
Gene scan statistic	10 (7)	38 (17)	87 (20)	135 (44)
Total	43 (15)	38 (17)	159 (32)	240 (64)

^a^Numbers in parentheses are counts for tests with genome-wide empirical *p*-values < 0.05.

We also compared the region-based statistics (global, Schaid, James minP) and scan statistic results for a list of 62 genes reported to be associated with HDL (17 genes), LDL (25 genes), or TG (20 genes) according to previously published reports [8,13,14]. In Table 3 we report the genes identified as significant by either the scan statistic (regional *p*-value < 10^-3^) or at least one of the region-based tests (asymptotic *p*-value < 0.0002 for analysis based on the principal components). In most cases, the genes identified by the region-based tests were also found by the scan statistic. In some cases, a scan statistic region from the pruned data did not overlap with a gene, but the results from the unpruned data did, as indicated in the rank column. On the other hand, scan statistics detected some candidate genes not identified by any of the region-based tests.

**Table 3:** Region-based tests of candidate genes for lipid phenotypes

	Gene-based analysis (*p*-values)^a^					Scan statistic analysis			
		
Lipid Gene	Chr.	No. SNPs (No. PCs)	Global LR test	Schaid test	James min *P *test	No. SNPs	Region *p*-value	GW rank	Empirical GW *p*-value^b^
**HDL**									
*CETP*	16	7 (3)	**7.96 × 10^-28^**	**3.32 × 10^-20^**	**3.81 × 10^-16^**	22	4.72 × 10^-17^	2	**<1.0 × 10^-5^**
*LPL*	8	5 (3)	**7.54 × 10^-7^**	**8.95 × 10^-7^**	**8.52 × 10^-6^**	12	1.06 × 10^-8^	6	**9.42 × 10^-4^**
*ABCA1*	9	52 (14)	**1.67 × 10^-6^**	0.15	1.12 × 10^-3^	16	2.51 × 10^-8^	10	**1.50 × 10^-3^**
*HERPUD1*	16	2 (2)	0.36	0.15	0.45	22	4.72 × 10^-17^	2	**<1.0 × 10^-5^**
*SLIT1*	10	47 (10)	4.27 × 10^-4^	**1.87 × 10^-4^**	0.02	6	6.15 × 10^-4^	197	0.31
*LIPG*	18	1 (1)	0.29	0.29	0.29	39	7.81 × 10^-26^	1	**<1.0 × 10^-5^**
*ACAA2*	18	5 (2)	0.67	0.42	0.61	39	7.81 × 10^-26^	1	**<1.0 × 10^-5^**
**LDL**									
*PSRC1*	1	1 (1)	**2.43 × 10^-25^**	**2.43 × 10^-25^**	**1.21 × 10^-25^**	3	4.20 × 10^-6^	218^c^	**0.02**
*LDLR*	19	5 (2)	**2.67 × 10^-5^**	**3.80 × 10^-5^**	**9.91 × 10^-6^**	15	1.82 × 10^-8^	14	**1.10 × 10^-3^**
*APOB*	2	10 (4)	**2.33 × 10^-11^**	**5.41 × 10^-11^**	**2.06 × 10^-9^**	17	9.40 × 10^-10^	7	**2.22 × 10^-4^**
*HMGCR*	5	5 (2)	5.52 × 10^-4^	**1.09 × 10^-4^**	1.38 × 10^-3^	NA^d^	NA	NA	NA
*BCAM*	19	1 (1)	0.09	0.09	0.09	18	6.09 × 10^-11^	3	**4.69 × 10^-5^**
**TG**									
*TBL2*	7	3 (2)	**8.38 × 10^-14^**	**2.78 × 10^-14^**	**6.81 × 10^-12^**	7	4.64 × 10^-10^	106^c^	**4.75 × 10^-5^**
*LPL*	8	5 (3)	**3.23 × 10^-11^**	**1.70 × 10^-11^**	**1.84 × 10^-9^**	24	1.27 × 10^-16^	3	**<1.0 × 10^-5^**
*GCKR*	2	4 (2)	**8.98 × 10^-13^**	**8.17 × 10^-10^**	**2.46 × 10^-11^**	6	5.51 × 10^-6^	40	**0.013**

^a^For tests in regression analysis of principal components (PCs). *p*-Values < 2 × 10^-4 ^are in bold.

^b^The empirical *p*-value is the number of permutation regions with *p*-values smaller than the observed regional *p*-value divided by 10,000 *n*, where *n *is 240 for HDL, 243 for LDL, or 204 for TG. *p*-Values < 0.05 are in bold.

^c^Rank from the scan statistic analysis using unpruned genotype data

^d^NA indicates that the regional *p*-value was greater than the threshold 10^-3^.

## Conclusion

We consider chromosomal regions as the unit of analysis, rather than SNPs, so that the dimensionality problem is reduced at the genome-level. However, when using the scan statistic, the issue of criteria for genome-wide significance is difficult to address because the dimension of the problem is not well defined with testing of many possible overlapping regions consisting of different window sizes. Here we used positional permutation of *p*-values to obtain genome-wide regional *p*-values.

In using the statistically defined regions without referring to the top SNPs, it appears that although we missed a number of significant single SNPs, we also identified many additional regions not found as SNP-database regions. The scan-statistic approach could also be used as a first stage in GWAS analysis, followed by within-region fine-mapping and/or direct sequencing. Once a region is detected, both approaches require follow-up with additional analyses to assess specific SNP variation within a region.

## List of abbreviations used

FHS: Framingham Heart Study; GEE: Generalized estimating equations; GWAS: Genome-wide association study; HDL: High-density lipoprotein; LD: Linkage disequilibrium; LDL: Low-density lipoprotein; SNP: Single-nucleotide polymorphism; TG: Triglycerides.

## Competing interests

The authors declare that they have no competing interests.

## Authors' contributions

JLA implemented the scan statistic analysis and drafted the manuscript. YJY designed and conducted the gene-based analyses. DW carried out the single-SNP analysis, including quality control and comparison of genome-wide results. LS contributed to the conception and design. SBB conceived the study, and participated in its design and coordination. SBB and YJY helped to draft the manuscript. All authors read and approved the final manuscript.
